# John B. Goodenough’s Role in Solid State Chemistry Community: A Thrilling Scientific Tale Told by a French Chemist ^†^

**DOI:** 10.3390/molecules25246040

**Published:** 2020-12-21

**Authors:** Michel Pouchard

**Affiliations:** CNRS, University Bordeaux INP, ICMCB, UMR 5026, F-33600 Pessac, France; michel.pouchard@icmcb.cnrs.fr

**Keywords:** structure, bonding, physical properties, collective or localized electrons, exchange integral

## Abstract

In this tribute to John B. Goodenough I will describe how John’s talk on the metal-to-nonmetal transition of vanadium oxide VO_2_, presented at the Bordeaux Conference (September 1964) attended by inorganic chemists, metallurgists, crystallographers, thermodynamicists and physicists, provided a pioneering vision of interdisciplinary research to come. John gave a complete description of the paradigm on how the physical properties of a solid depend on its structure and bonding, by employing the chemical notions as local distortions and interatomic distances as well as the physics notions such as band width and the Hubbard on-site repulsion U. I will illustrate how inspiring John’s ideas were, by discussing the research examples of my own research group in the sixties-seventies. The fundamental approach of John B. Goodenough to Solid State Chemistry, leading particularly to lithium battery applications, is at the heart of the 2019 Nobel Prize awarded to John.

## 1. Introduction

Tuesday morning, 8 October 2019, my cell phone received a fantastic news that John B. Goodenough was awarded the Nobel Prize, the first one given to solid state chemistry. This was a joyful moment for all of his friends! Suddenly, I realized how profoundly a cell phone in my hand, a very common object these days, changed our everyday life thanks to the fast progress of research and technology in lithium batteries. On reflection, Pasteur’s famous words came to my mind: “There are science and applications of science, which are so intimately bound together than the tree and the fruits that it bears …” In this Special Issue dedicated to John, I would like to reminisce on the role played by John in the early development of solid state chemistry, the tree that can bear so many different fruits in the future.

## 2. The Beginnings of Solid State Chemistry

In the early 20th century, inorganic chemistry was essentially concerned with the discovery of new elements such as the noble gases (W. Ramsay), fluorine (H. Moissan), rare-earths (G. Urbain), and the radioactive elements, radium and polonium, (the Curie’s). These studies led to three Nobel prizes between 1904 and 1911. After the First World War, following the discovery of X-ray diffraction for the determination of crystal atomic structures (Laue, Bragg, Ewald), inorganic chemistry started dealing with more and more complex systems such as the inorganic core (Fe, Co or Mg) in biological molecules such like hemoglobin and metalloporphyrins (Perutz, Nobel Prize 1962). Simultaneously, quantum mechanics provided the foundation for the description of electronic structure and chemical bonding (De Broglie, Dirac, Heitler, Hund, Hückel, Mulliken). As early as 1939, Ewans elaborated a structural chemistry, which enables one to deduce chemical and physical properties from X-rays atomic structures. In the same year, Pauling published his famous textbook, “The nature of the chemical bond and the structure of molecules and crystals”, establishing the valence-bond approach to chemical bonding, for which he received the Nobel Prize in 1954. The molecular orbital approach to chemical bonding, an alternative to the valence bond approach, was subsequently developed by Mulliken, who received the Nobel Prize in 1966.

In the early 20th century, inorganic chemistry in France was carried out by three forerunners: C. Friedel, H. Moissan and H. Le Chatelier, later by Friedel’s students, G. Urbain and A. Chretien, by Moissan’s students, P. Lebeau and L. Hackspill, and by Le Chatelier’s student, G. Chaudron. They held prestigious Chairs at The Sorbonne, College de France and Engineering School institutions. At the end of The Second World War, inorganic chemistry was divided essentially in two areas, chemistry of solutions led by A. Chretien and metallurgy led by G. Chaudron; they disagreed totally on their visions of chemistry concerning the use of physical methods and applications. Chretien’s students, P. Hagenmuller and A. Deschanvres, and Chaudron’s student, R. Collongues, attempted to find some common grounds. As early as 1948, Chaudron had organized a series of International Symposia on the Reactivity of Solids. This series was rather limited in terms of topics and international participations, and gradually disappeared. 

In Germany, inorganic solid state chemistry was most recognized for its successes in synthesis, for example, in the area of transition metals of high oxidation states by W. Klemm and in Zintl-type phases between alloys and normal compounds. However, collaborations with physicists and application attempts remained poorly developed. In USA, solid state inorganic chemistry was essentially developed in the industrial laboratories such as Dupont, Exxon, IBM and General Electrics, strongly motivated by the applications and materials science. There were high-level studies in thermodynamics and non-stoichiometry by J. S. Anderson in United Kingdom, and those in extended defects of non-metallic solids by A. Magneli and S. Anderson in Sweden and by D. Wadsley in Australia, following the pioneering works in this defects in solid field (Frenkel, Schottky and Wagner). 

## 3. Bordeaux, September 1964:”Crystallization” of a Solid State Chemistry Community

In Spring 1960, Paul Hagenmuller organized, with his group, a long visit to the main inorganic chemistry laboratories in Germany (Klemm and Schäfer in Munster, Scholder in Karlsruhe, Glemser in Göttingen, Rabenau in Aachen). Four years later, he invited them as well as many foreign colleagues mostly from USA to Bordeaux for an important international symposium on the “transition metal oxides in solid state”. The scientific community was broadly chosen: physicists (P. Aigrain as an expert of semiconductors, F. Bertaut representing L. Néel, the future French Nobel Prize winner in physics, C. Guillaud etc.), French and German inorganic chemists, a large group of metallurgists (G. Chaudron, J. Bénard, P. Lacombe, A. Michel etc.), a few specialists of thermodynamics, crystallographers (S. Anderson, G. Blasse, E.W. Görter etc.) as well as materials chemists of principally from USA (J. B. Goodenough, A. Wold, R. Roy). 

**Figure 1:**
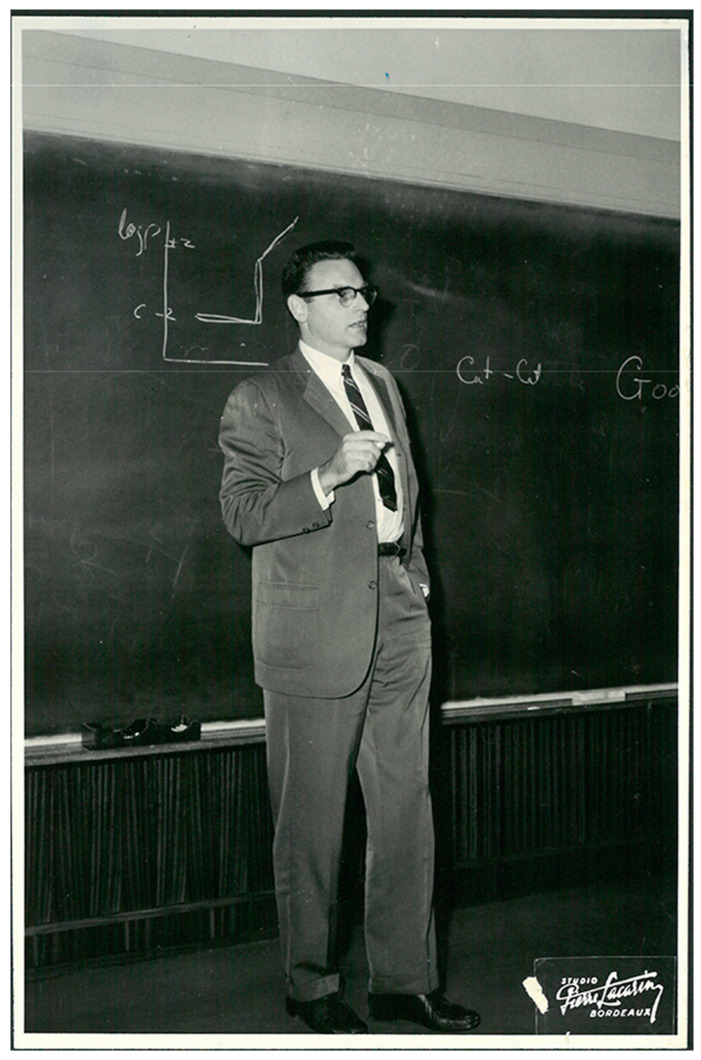
J.B. Goodenough at the Bordeaux-Congress of 1964 presenting the metal-non-metal transition (structural as electronic) in the vanadium di-oxide VO_2_.

In front of this mixed Assembly John Goodenough gave an unforgettable lecture on the metal/non-metal transition in vanadium oxide, VO_2_ (Figure 1). This phase transition had been observed earlier, just before Second World War, by the metallurgist A. Michel, who attended the symposium. John first introduced the small range of composition homogeneity for this oxide (VO_2−δ_), close to the Magneli-oxide series V_n_O_(2n−1)_, with crystallographic shear planes beautifully imaged by high-resolution electron microscopy.

For the specialists of thermodynamics, John recalled the temperature vs. oxygen partial pressure conditions for the H_2_/H_2_O or CO/CO_2_ gas-phase mixture needed for the synthesis, related to the famous Chaudron’s diagram. Secondly, John turned to the crystallographers, showing the crystal symmetry breaking at the transition, from tetragonal P4/mnm to monoclinic P2_1_/c, then to the chemists, showing how the VO_6_ octahedron undergoes a large distortion in the monoclinic form. To the physicists, he spoke the band structure language, with the band splitting along the distortion from a π*(t_2g_) triplet into an occupied singlet b_2g_^1^ and an empty doublet e_g_^0^. He underlined the periodicity doubling along the *c* axis, as expected in this Peierls-type transition, following the π*(b_2g_^1^) band splitting into two split narrow bands, of which only the lowest one is doubly occupied (b_2g_^2′^). John showed to the chemists how this splitting modifies the electron distribution along the chain of vanadium atoms, increasing the electron density and bonding character in the V-V pairs, decreasing the density (of antibonding character) between the vanadium pairs. Everybody understood that the different descriptions correspond actually to the same physical reality, but a different picture emerged for different eyes, with everybody finding one’s own place. As for myself, working at that moment on the vanadium oxide bronzes, John’s talk was a revelation, a guideline for my future as a scientist. I often recall this magic moment. When growing single crystals by cooling a crucible filled with many components, one often finds crystallization into separate binary, sometimes ternary, compounds. However, introducing an appropriate seed induces the formation of a beautiful multi-component single crystal, in which all components find their own and desired places in a perfectly ordered three-dimensional arrangement. *In September 1964, John Goodenough was such a seed for the future development of the solid state community.*

## 4. Solid State Chemistry and Localized Versus Delocalized Electrons

In the early 1950s, transition-metal oxides were believed to possess strong ionic bonding and hence are insulators that can be described by crystal-field theory and by magnetic super-exchange or Zener-type double-exchange. However, the band structure description of insulating oxides (e.g., NiO) found that they possess partially-filled bands, hence predicting that they are metals in contradiction to experiment. In the late 1950s, sodium tungsten bronzes Na_x_WO_3_, discovered one century ago, were found to be a metal. Similarly, the mixed-valent manganite La_(1−x)_ Sr_x_ MnO_3_ was found to be a ferromagnetic metal. In fact, two opposite theories existed for the description of the outer electrons of solids: the crystal-field and band theories. The crystal-field theory describes localized electrons at discrete atomic positions with a long residence time τ. With a short residence time τ, each electron feels the periodic potential of the crystal and becomes delocalized (collective). The latter is the case for s and p electrons, but the former for 4f electrons. d electrons lie in-between the two, localized in some crystals but delocalized in others. At the beginning of the sixties John explored this difference, especially for the AMO_3_ perovskite and related structural classes of solids. These studies led to many review papers, book chapters/series and more specific articles [1,2,3,4,5,6,7,8,9,10,11].

There are two fundamental parameters to consider in understanding the metal vs. insulator behaviors of solids; the transfer energy b^ca^ between cationic and anionic orbitals and the on-site Hubbard U parameter separating the formal valences between the +m and +(m-1) oxidation states of a cation. For the free ion, U can be expressed as the energy difference between the successive ionization energies EI^m+1^−EI^m^ corrected by the electron-hole Coulomb attraction. The competition between b^ca^ and U, or the b^ca^/U ratio, sets the boundary for the localized vs. delocalized character of the outer d-electrons. Besides, both parameters depend on the covalency: b^ca^ increases with covalency (and so does the band width), while U decreases with covalency (so does the Racah B parameter of the molecular chemists). Electrons become delocalized above a critical value b_c_^ca^ and metallicity sets in just later (b_m_), when the occupied and unoccupied states (bands) overlap. Thus, in the early 1960s, J. B. Goodenough proposed a phenomenological diagram, b^ca^ = f (Z, S), to classify the transition metal oxides of perovskite structure with localized vs. delocalized d-electrons in terms of the atomic number Z and spin S of the transition metal ion.

The AMO_3_ perovskite structure consists of corner-sharing MO_6_ octahedra, involving thus a cubic close-packing arrangement of oxygen atoms with one quarter of which replaced by the large A cation at the body-center positions. With the ideal ionic radii R_A_, R_M_ and R_O_ of A, M and O that allow the cations and anions to be in contact for the optimal Coulomb interactions, the geometric tolerance factor t,

$$t=\frac{\left(R_{A}+R_{O}\right)}{\sqrt{2}(R_{M}+R_{O})},$$

becomes 1. Many parameters can modify the values of ionic radii:

(i) Coordination number, temperature, pressure, including chemical pressure, i.e., substitution with an ion of different radius.
(ii) Spin equilibrium or transition. For example, a d^6^ cation with three possible configurations, low-spin t^6^e^0^, high-spin t^4^e^2^, and sometimes intermediate-spin t^5^e^1^, in a Jahn-Teller distorted site or in metallic compounds (t^5^σ*^1^), as discussed in details by John in his articles on LaCoO_3_ and LaNiO_3_ (9,10).
(iii) Charge disproportionation, as observed by Takano et al. [12] in CaFeO_3_ synthesized under oxygen high-pressure (Fe^4+^ with t^3^e^1^, giving rise to Fe^5+^ with t^3^e^0^ plus Fe^3+^ with t^3^e^2^) and extensively discussed by John.
(iv) Insulator to metal transition, often associated with a large decrease in the cell volume, as found for hole-doped divalent cuprates.

Let us consider one example illustrating the point (i): silica and silicates have corner-sharing SiO_4_ arrays at ambient pressure. Under geological pressure in the deep mantle, the (R_M_/R_O_) ratio strongly increases due to oxygen polarizability and compressibility; for the same number of inner electrons [10], the volume of the oxide ion is approximately thirty times greater than silicon. In these conditions, silicon becomes octahedrally coordinated in the ilmenite and perovskite structures. Conversely, a cation such as Fe^2+^ becomes smaller under pressure by changing its configuration from t^4^e^2^ into low-spin t^6^e^0^. As a consequence, the atomic and electronic structures strongly influence all properties of solids.

AMO_3_ oxides may also adopt the hexagonal close-packing of oxygen, in which face-sharing octahedral chains develop along the *c* axis (t > 1). This less dense hexagonal form transforms naturally into the cubic form under pressure. In several general articles, John discussed such transitions through successive well-ordered face- centered cubic and hexagonal close-packing sequences, giving rise to various intermediate phases named “poly-types”, such as 4-H or 9-H in the oxides BaTiO_3_ and BaRuO_3_, respectively, and in AMCl_3_ chlorides. These early studies allowed John to classify structures on the basis of inter-cationic Coulomb repulsions (charge, distance) related to the covalent mixing and the structural pattern of corner-, edge-, or face-sharing. For edge-sharing entities, he defined a critical value (R_C_) separating the localized and collective electrons. An important consequence concerns the MO_2_ series: with the earliest 3d elements (low-covalent mixing and highest charge), only the rutile structure with two common edges is allowed. The six common edges of the 2D layered brucite-type structure was an important challenge for the electrode-battery materials in sodium cobalt bronzes by Fouassier et al. [13], Delmas et al. [14], and finally Tarascon et al. [15] due to reduced cationic charges in the mixed-valent bronzes.

For the t values smaller than unity, the twelve coordination site A of cubic AMO_3_ oxides is too roomy for the mid and late rare-earth cations. To accommodate the small cations at the A site, the pristine cubic cell adjusts by a cooperative rotation of the MO_6_ octahedra along the [111] axis for a R3m rhombohedral symmetry, and further along the [110] axis for an orthorhombic Pbnm symmetry. The bending angle in M-O-M bonds decreases smoothly with t to preserve the A-O contacts, typically until an eight-coordination environment is achieved. A few examples of the evolutions along the 3d series with A = Ln^3+^ or Sr^2+^ and Ca^2+^ are:

- For the largest M^3+^ cation (Ti) of t_2g_^1^e_g_^0^ configuration with b^ca^ < b_c_ < b_m_, only the orthorhombic Pbnm structure is accessible. The M-O-M bending increases along with the lanthanide series contraction, the band width decreases, and the band gap increases from 0.2 eV for La up to 1.2 eV for Y.
- For smaller cations (Cr^3+^ and Fe^3+^), the increase in b^ca^ is not significant enough to overcome the effect of U, and the strong spin exchange interactions make these compounds antiferromagnetic insulators.
- For the intermediate lanthanum manganite LaMnO_3_ and its high-spin t_2g_^3^e_g_^1^ configuration, one can predict either an insulating Jahn-Teller distorted situation as well as a metallic state (t^3^σ*^1^ for b^ca^ overcoming b_m_). The former configuration was accepted early, but not totally understood until John’s enlighting explanation [7] based on the alternating (x^2^ − y^2^) and (z^2^) atomic orbitals along both [100] and [010] axes, in agreement with the unusual ratio c/a < 1. Besides, such orbital ordering was among the most spectacular illustrations of the Goodenough-Kanamori rules for ferromagnetic super-exchange. At this point, it is interesting to discuss briefly the case of Sr ferrates containing Fe^4+^ ions with the same d^4^ configuration. b^ca^ is larger for Fe^4+^ than for Mn^3+^ cations, allowing the t^3^σ*^1^ metallic configuration for Fe in SrFeO_3_. However, with the smaller Ca cation and the associated orthorhombic distortion, as well as with the decrease in b^ca^ and band width, a metallic state is not allowed anymore for CaFeO_3_. As mentioned above, a new type of ordering occurs at low temperature, with a partial disproportionation of two Fe^4+^ into Fe^5+^ + Fe^3+^. This interesting new behavior, observed by ^57^Fe Mossbauer spectroscopy, was largely discussed by John and Mikio Takano [16]. A few years later, we were able to confirm this partial disproportionation by comparing their data to ours in La_2_LiFeO_6_ (with a much larger negative value of the isomer-shift δ = −0.41 mm.s^−1^) [17].
- LaNiO_3_ (R3c) was the only rare-earth nickelate known up to the seventies. Its metallicity was proven only in 1965 by Goodenough and Racah [9], which results from the low-spin t_2g_^6^e_g_^1^ configuration and b^ca^ > b_m_, which induces the involvement of the t_2g_^6^σ*^1^ configuration.

The AMO_3_ perovskites allow anionic vacancies different from ordered defects of Magneli and Wadsley type in crystallographic shear planes and so do the ReO_3_-type oxides, which are related to the AMO_3_ perovskites except that the large central A site is empty. Of particular interest was John’s description of the Brownmillerite calcium ferrite mineral Ca_2_Fe_2_O_5_ (2), i.e., CaFeO_2.50_, exhibiting a large number of vacancies compared with the pristine AMO_3_ perovskite: half the Fe^3+^ ions occupy the distorted corner-sharing octahedra with the remaining half occupying the corner-sharing tetrahedra, and the planes containing these Fe^3+^ ions alternate. We will discover in the following how important this description by John was. 

The contributions of John Goodenough to solid state chemistry are too numerous to summarize in a few lines. His contributions broadened the vision of every solid state chemist by providing them with the crystal-field description of the ionic solids, the molecular description of the chemical bond, and the physics view of the delocalized outer-electrons.

## 5. Solid State Chemistry, Goodenough’s Heritage

Every solid state inorganic chemist of my generation (young researchers in the 1960s to 1970s) has been influenced, or more often inspired, by John’s ideas, which were guidelines for explaining or predicting new properties for new materials. Since it would be unrealistic to give an exhaustive list of his ideas and concepts, I will limit my reminiscences to those that greatly influenced the thinking and development of my research group in the 1960s to 1970s.

### 5.1. Electrical Conductivity of Tungsten Bronzes and the Goodenough-Sienko Debate

For Mike Sienko, the metallic conductivity observed in tungsten oxide bronzes resulted from the direct t_2g_−t_2g_ overlap of W 5d orbitals. In contrast, John proposed an indirect overlap through the 2p_π_ orbitals of oxygen, on the basis of the insulating character of the double perovskite Sr_2_MgReO_6,_ (W^5+^ and Re^6+^ are isoelectronic with 5d^1^). However, other explanations are possible due to the tetragonal distortion in the latter as well as the different nature and size of all ions involved. In a contribution to this debate, J. P. Doumerc [18] replaced some oxygen atoms by fluorine, more electronegative than oxygen, which should decrease the conductivity in an indirect mechanism. He showed experimentally this to be the case, but it was argued not to be an absolute proof: actually, since the Anderson-type anionic disorder (O and F) would lead to a similar effect. This issue was resolved many years later to the satisfaction of both John and Mike! For Mike, for the lowest values of x in Na_x_WO_3_, because the Fermi level E_F_ lies close to the center of the Brillouin zone, where the mixing between the t_2g_(W) and 2p_π_(O) orbitals is symmetry-forbidden. For John, for much larger x values (for which the bronze is fully metallic) because the Fermi level lies far away from the zone center, where the cationic and anionic orbital mixing takes place. We were relieved to find this friendly solution!

### 5.2. Ternary Tungsten Oxides MWO_4_ (M = Al, Cr, Ga) of W^5+^ Ions

Following John’s lecture in 1964, the metal/non-metal transition of VO_2_ led to numerous developments in USA (IBM) and in France (Orsay, Bordeaux) generating new collaborations between physicists and chemists, and to the discovery of new intermediate phases such as Marezzio et al.’s [19]. In this chromium-doped monoclinic variety of VO_2_ (C2/m), every second chains of V^4+^ ions have dimerized V^4+^ pairs, as in what would be an ordered phase including the high and low-temperature forms of VO_2_. Another way to stabilize the W^5+^ ions is to pair them in one chain and replace the W^5+^ ions of the other chain with trivalent d^0^ cations (e.g., Al^3+^), d^10^ cations (e.g., Ga^3+^) or even a spherical d^3^ ion (e.g., Cr ^3+^). The small W-W pairs were found to be particularly stable up to 1000 °C [20]. 

### 5.3. Nickelates of Perovskite Structural Type LnNiO_3_ (Ln = rare-earth, La-Lu, Y)

In 1964, LaNiO_3_ was the only known rare-earth nickelate. Five years later, in Bordeaux, G. Demazeau began his PhD studies, introducing in our laboratory the Belt-type high-pressure synthesis facilities adapted to produce high oxygen pressure by decomposing KClO_3_ in situ. Among a large number of transition metal oxides, which system should one study? Perovskite, of course, for their high stability under pressure! The nickelate system appeared also as an obvious choice, with immediate questions to raise. Does a complete series from La up to Y exist as discovered for Ti^3+^ under reducing conditions in Germany in the late sixties? Low-spin trivalent nickel is much smaller than trivalent titanium. Do all nickelates LnNiO_3_ possess low-spin Ni^3+^? Are they metallic? In 1972 we obtained all the answers [21,22]. The series was complete, from the rhombohedral and metallic phase with large La to the orthorhombic and insulating phase with smallest rare-earths Lu and Y. As predicted, the increased tilting of the NiO_6_ octahedra together with the decrease in rare-earth size narrows the σ*^1^ band, with b^ca^< b_m_. In addition, an antiferromagnetic order appeared at low temperature. Twenty years later, at IBM San Jose, Torrance and Lacorre [23,24] were able to draw the first phase diagram of the insulator-to-metal transition temperature T_t_ and the Néel temperature T_N_ for the LnNiO_3_ family. Today, such a diagram is found in solid state chemistry textbooks. A strontium-doped member of this nickelate family, Nd_1-x_Sr_x_NiO_3_, reduced by H_2_ in a layered thin-film oxide AMO_2_, was recently found to be a superconductor, stimulating new reflections on the superconductivity mechanisms [25].

### 5.4. Long-Range Ordering of Planar Defects in Some Non-Stoichiometric Perovskite-Type Ferrites 

To my knowledge, John Goodenough was the first to propose the description of Ca_2_Fe_2_O_5_ as an ordered succession of corner-sharing octahedron layers and corner-sharing chains of tetrahedra, arranged also in parallel layers. Both coordination types, approximately octahedral and approximately tetrahedral, agree with the spherical character of high-spin Fe^3+^ (d^5^, S = 5/2), insensitive to the crystal field symmetry. For such a high extent of oxygen vacancies (0.50 per AMO_3_ formula unit) with all A sites occupied, any crystallographic shear mechanism can be envisaged. During his PhD studies in Bordeaux (1975), Grenier et al. imagined that all AFeO_3−δ_ non-stoichiometric compositions with 0 < δ < 0.50 can be described with the same type of layer alternation of octahedral (O) and tetrahedral (T) coordinations, with various sequences such as OOTOOT (δ = 0.33), OOOTOOOT (δ = 0.25), as well as inter-growth situations (OOTOTOOTOT, δ = 0.40). J.C. Grenier synthesized the solid solutions between CaFeO_2.50_ and CaTiO_3_ as well as LaFeO_3_, and confirmed the proposed non-stoichiometry-based structural model by X-ray diffraction, high-resolution electron microscopy and ^57^Fe-Mössbauer spectroscopy [26,27,28]. Such a type of layered, ordered planar defects is associated with the electron configuration of the cation Fe^3+^ implying the octahedral and tetrahedral symmetries for the basic polyhedra. Ten years later, oxides of other d-electron configurations like d^4^, d^7^ and d^9^ were prepared and studied. In particular, d^9^ ions led to the cuprates family, based on layers of square-pyramidal (C_4v_) and square-planar (D_4h_) polyhedra arrangements of Cu^2+^ ion.

### 5.5. Trivalent Cuprates, a Precursor to High-T_C_ Superconductors

Back in 1970, trivalent cuprates of perovskite structure were not known, despite the similarity between Cu^3+^ and Ni^2+^. However, a decade earlier, Klemm, and subsequently Hoppe, synthesized MCuO_2_ phases (M = Na, K) with Cu^3+^ in square-planar coordination, which exhibit diamagnetism and insulating behaviors. During John’s visit to Bordeaux for a few months in the late sixties, we had a lot of discussions with him about the influence of bond covalency on the band width and electron delocalization and also about the similarity between d^1^ (one electron) and d^9^ (one hole) systems. A strong covalent character meant the presence of highly destabilized states at the top of the filled 3d band, and hence a large energy stabilization by hole doping. This realization led to the associated question: is covalency strong enough to overcome the Jahn-Teller instability and induce metallicity? The previous success in synthesizing the LnNiO_3_ series under high oxygen pressure was an argument for attempting the synthesis of new Cu^3+^ oxides. We chose three compositions: (i) LaCuO_3_ for its expected 3D metallicity, (ii) LaSrCuO_4_, the first member of the Ruddlesden-Popper series of 2D perovskite-related structures, so as to evaluate the Jahn-Teller vs. metallicity competition, and (iii) La_2_Li_1/2_Cu_1/2_O_4_ to evaluate the influence of Li chemical pressure, an additional effect to prepare the S = 0 low-spin d^8^ configuration of Cu. All these synthesis attempts were successful with physical properties as predicted. These results were published in 1972-73 [29,30,31], with the participation of John Goodenough, and that of Sir Nevill Mott (for the last paper), who was awarded the Nobel Prize in physics a few years later (1977). Ten years later, these results were enriched by the study of Raveau et al. on mixed-valent Cu^2+^/Cu^3+^ cuprates [32]. This work was followed by the discovery of superconductivity at 35 K in 1986 by Bednorz and Müller in this system [33], for which they received the Nobel Prize in 1987.

Before concluding on John’s achievements during the seventies, I would like to mention another important areas in which fundamental science and applications are intimately linked: Na^+^ cationic conductivity in phospho-silicate oxides with tunnel structures (NASICON: Natrium-Super-Ionic-CONductors), as well as O^2−^ anionic conductivity in La_1−x_Sr_x_GaO_3−y_ gallates for solid oxide fuel cell (SOFC) applications, and solar energy simultaneous capture and storage by wavelength-selective photo-anodes, generating hydrogen from water by photo-electrolysis (Honda-type experiment). 

## 6. Concluding Remarks

In summary, John Goodenough greatly influenced the thinking of the solid state chemists and played a crucial role in the early development of solid state chemistry. He recognized the interdisciplinary nature of solid state chemistry and clearly demonstrated the paradigm that, for understanding and getting useful applications out of solid state chemistry, it is crucial to know the interrelationship between structure, bonding and properties. I would like also to point out more recent contributions of physics (P.G. de Gennes, Nobel Prize in 1991) for the soft matter and those of theoretical chemistry (R. Hoffmann, Nobel Prize in 1981, and his active disciples) in enriching this paradigm.
